# Emerging Role of Non-Coding RNAs in Senescence

**DOI:** 10.3389/fcell.2022.869011

**Published:** 2022-07-05

**Authors:** Soudeh Ghafouri-Fard, Tayyebeh Khoshbakht, Bashdar Mahmud Hussen, Aria Baniahmad, Wojciech Branicki, Mohammad Taheri, Ahmad Eghbali

**Affiliations:** ^1^ Department of Medical Genetics, School of Medicine, Shahid Beheshti University of Medical Sciences, Tehran, Iran; ^2^ Phytochemistry Research Center, Shahid Beheshti University of Medical Sciences, Tehran, Iran; ^3^ Department of Pharmacognosy, College of Pharmacy, Hawler Medical University, Erbil, Iraq; ^4^ Center of Research and Strategic Studies, Lebanese French University, Erbil, Iraq; ^5^ Institute of Human Genetics, Jena University Hospitals, Jena, Germany; ^6^ Malopolska Centre of Biotechnology, Jagiellonian University, Krakow, Poland; ^7^ Urology and Nephrology Research Center, Shahid Beheshti University of Medical Sciences, Tehran, Iran; ^8^ Anesthesiology Research Center, Mofid Children Hospital, Shahid Beheshti University of Medical Sciences, Tehran, Iran

**Keywords:** lncRNA, miRNA, senescence, expression, biomarker, epigenetics

## Abstract

Senescence is defined as a gradual weakening of functional features of a living organism. Cellular senescence is a process that is principally aimed to remove undesirable cells by prompting tissue remodeling. This process is also regarded as a defense mechanism induced by cellular damage. In the course of oncogenesis, senescence can limit tumor progression. However, senescence participates in the pathoetiology of several disorders such as fibrotic disorders, vascular disorders, diabetes, renal disorders and sarcopenia. Recent studies have revealed contribution of different classes of non-coding RNAs in the cellular senescence. Long non-coding RNAs, microRNAs and circular RNAs are three classes of these transcripts whose contributions in this process have been more investigated. In the current review, we summarize the available literature on the impact of these transcripts in the cellular senescence.

## Introduction

Living organisms encounter a gradual weakening of functional features. This phenomenon is called senescence or biological aging. This word may refer to either cellular senescence or to organismal senescence, the latter being characterized by an increase in death rate and/or a reduction in fertility parallel with increase in age, at least in the later portion of the life cycle of an organism. Cellular senescence is a process that is principally aimed to remove undesirable cells by prompting tissue remodeling (Muñoz-Espín and Serrano, 2014). This process induces tissue remodeling *via* three consecutive processes: a constant proliferative arrest; a secretory phenotype that leads to recruitment of immune cells and induces changes in the extracellular matrix; and recruitment of neighboring progenitors that activate tissue repopulation (Muñoz-Espín and Serrano, 2014). Cellular senescence has a role in morphogenesis and patterning in the course of embryogenesis through removal of temporary structures and regulation of the relative quantities of diverse cell populations. This process is also regarded as a defense mechanism induced by cellular damage. In the course of oncogenesis, senescence can limit tumor progression. However, senescence participates in the pathoetiology of several disorders such as fibrotic disorders, vascular disorders, diabetes, renal disorders and sarcopenia. While in the initial phases of these pathologies, senescence can limit the fibrotic responses and trigger immune responses that clear the injured cells, at advanced phases, accumulation of senescent cells can lead to aggravation of the pathological manifestations (Muñoz-Espín and Serrano, 2014).

Diverse mechanisms have been found to affect cellular senescence. DNA damage or telomere shortening can induce DNA damage response which can activate senescence. Moreover, secretion of high quantities of certain proteins such as chemokines, pro-inflammatory cytokines and matrix metalloproteinases by senescent cells can activate this process in other cells (Sławińska and Krupa, 2021).

Recent studies have revealed contribution of different classes of non-coding RNAs in the cellular senescence. Long non-coding RNAs (lncRNAs), microRNAs (miRNAs) and circular RNAs (circRNAs) are three classes of these transcripts whose contributions in this process have been more investigated. These three classes of transcript can regulate gene expression through different mechanisms. While miRNAs mostly act at post-transcriptional level through binding with mRNAs and inducing mRNA degradation or suppressing translation, lncRNAs can act at different levels to affect gene expression. Both lncRNAs and circRNA can act as molecular sponges for miRNAs to influence bioavailability of miRNAs and release miRNA targets from their suppressive effects. In addition to this “Decoy” function, lncRNAs can control nuclear hormone receptor abundance or telomere length, or can modulate expression of tumor suppressors by influencing specific stages of their transcription or translation (Dianatpour and Ghafouri-Fard, 2017; Ghafouri-Fard et al., 2020). Expression of these transcript can be modulated by DNA methylation (Ji et al., 2020).

Notably, therapeutic targeting of miRNAs and lncRNAs has been recently suggested as a striking way for management of several disorders with antisense oligonucleotides and small interfering RNAs being the most promising modalities in this regard (Winkle et al., 2021). In the current review, we summarize the available literature on the impact of these transcripts in the cellular senescence.

## LncRNAs and Senescence

Expressions of lncRNAs have been found to be altered during the course of senescence. Moreover, a number of lncRNAs can induce or de-activate cellular senescence. TERRA is an example of lncRNAs contributing in cellular senescence (Grammatikakis et al., 2014). This lncRNA inhibits telomere elongation. Most notably, TERRA lncRNA is transcribed from telomere, a region which has been supposed to be transcriptionally silent. Inhibition of elongation of telomeric RNA is associated with the existence of several copies of the telomere UUAGGG repeat in the TERRA transcript (Porro et al., 2014) which offers TERRA a competitive inhibitory effect for TERT (Redon et al., 2010).

Expressions of ANRIL and Sirt1-AS have been shown to be down-regulated, while expression of miR-181a has been found to be increased in aging vascular smooth muscle cells (VSMCs). Functional studies in young and aging VSMCs have shown that up-regulation of ANRIL leads to down-regulation of miR-181a, up-regulation of Sirt1-AS and enhancement of cell viability and suppression of senescence in VSMCs. miR-181a directly targets both ANRIL and Sirt1-AS transcripts. Besides, the up-regulation of ANRIL is able to suppress cell cycle arrest and decrease activity of p53-p21 pathway (Tan et al., 2019a).

Another study in cervical cancer cell lines has shown up-regulation of ANRIL and down-regulation of miR-181a-5p. Short hairpin RNA-mediated silencing of ANRIL has led to inhibition of cell proliferation, invasiveness, migratory potential and enhancement of apoptosis and senescence. This study has also confirmed direct interaction between ANRIL and miR-181a-5p. Notably, TGFβI has been found to be targeted by miR-181a-5p (Zhu et al., 2019a).

**FIGURE 1:**
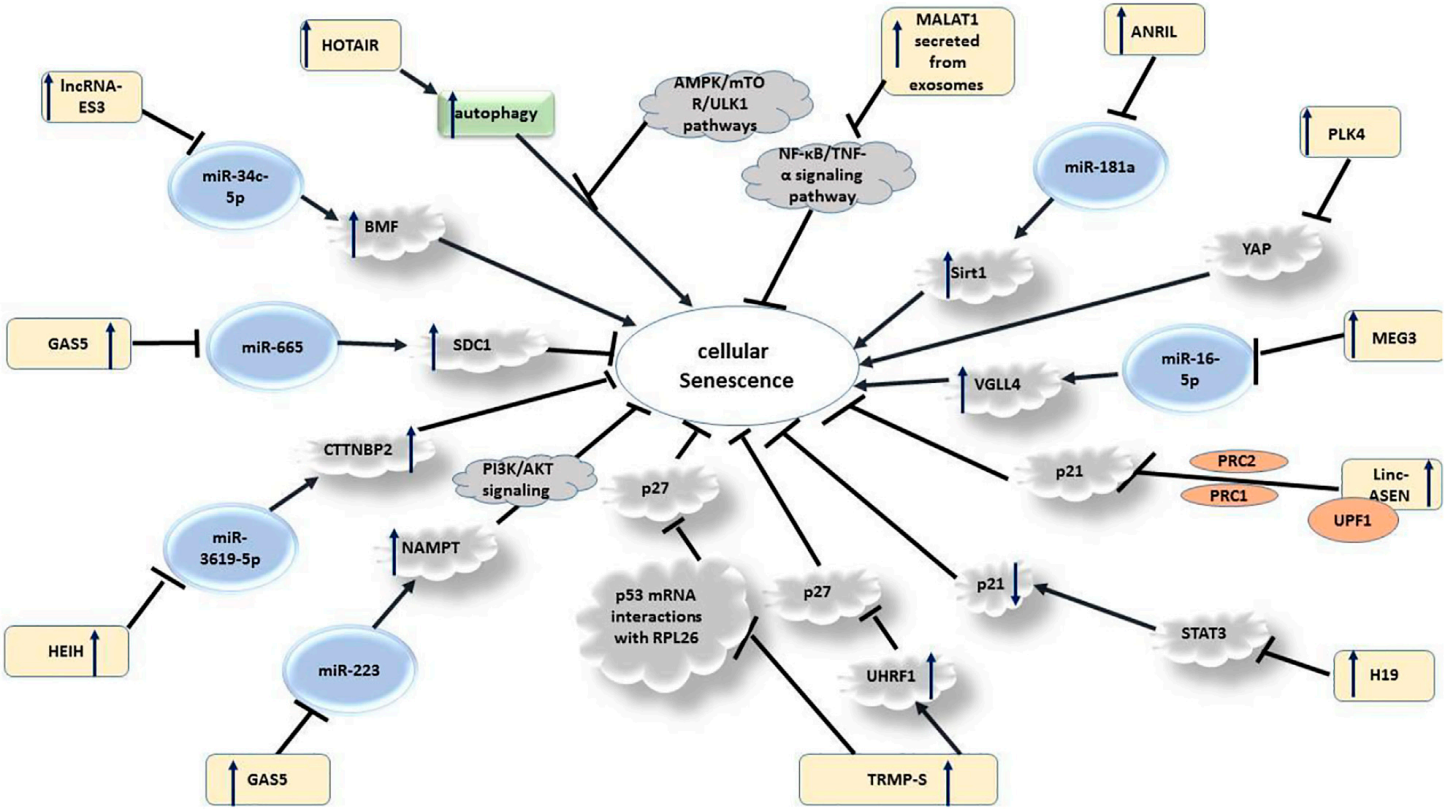
Summary of the role of lncRNAs in senescence.

LncRNAs might also regulate senescence process of endothelial progenitor cells (EPCs), a group of cells with potential for repair of injured endothelial cells. However, EPC senescence might lead to the failure in EPC function. GAS5 has been found to sponge miR-223, thus decreasing its expression. GAS5 silencing suppressed proliferation of EPCs and increased their senescence through counteracting with miR-223-mediated inhibition of NAMPT expression. Therefore, GAS5 can regulate proliferation and senescence of EPCS through modulation of the PI3K/AKT signaling (Yao et al., 2019). Another study has indicated that suppression of miR-665 by GAS5 modulates VSMC senescence through down-regulating SDC1 (Chen et al., 2021a). GAS5 has also been shown to be differentially expressed between young and old mouse brain samples, indicating its putative impact on senescence and brain aging. Expression of GAS5 has been significantly higher in the hippocampus region of old mouse compared with young mouse. In the hippocampus-derived HT22 cell line, GAS5 is localized in the nucleoplasm and cytoplasm. Functionally, GAS5 could inhibit cell cycle progression and induce apoptosis. Based on the results of RNA-Seq analyses, GAS5 can regulate expression of genes participating in cell proliferation. Moreover, GAS5 interacting proteins have been shown to be involved in senescence-associated disorders (Wang et al., 2021a). Figure 1 shows lncRNAs that participate in the senescence process.

Tano et al. have investigated the noncoding transcriptome of hepatocytes and cardiomyocytes in young and adult mice. Through this approach, they could isolate 62 lncRNAs associated with juvenility with selective expression in these two cell types. Gm14230 is one of these transcripts which has important roles for cellular juvenescence. Gm14230 knock-down has impaired cell growth and induced cellular senescence. Functionally, Gm14230 protects cellular juvenescence via recruitment of histone methyltransferase Ezh2 to Tgif2, thus suppressing the impact of Tgif2 in cellular senescence (Tano et al., 2019). GUARDIN is another lncRNA which can affect senescence. This lncRNA is an indispensable constituent of a transcriptional repressor complex containing LRP130 and PGC1α. In fact, this lncRNA serves as a scaffold to increase stability of LRP130/PGC1α heterodimers and their recruitment on the FOXO4 promoter. GUARDIN knock-down leads to destabilization of this complex and subsequent increase in FOXO4 expression. This also increases expression of p21, thus inducing cellular senescence. Notably, expression of GUARDIN is increased by rapamycin, a substance that inhibits senescence. Finally, FOSL2 can repress expression of GUARDIN (Sun et al., 2020).

**FIGURE 2:**
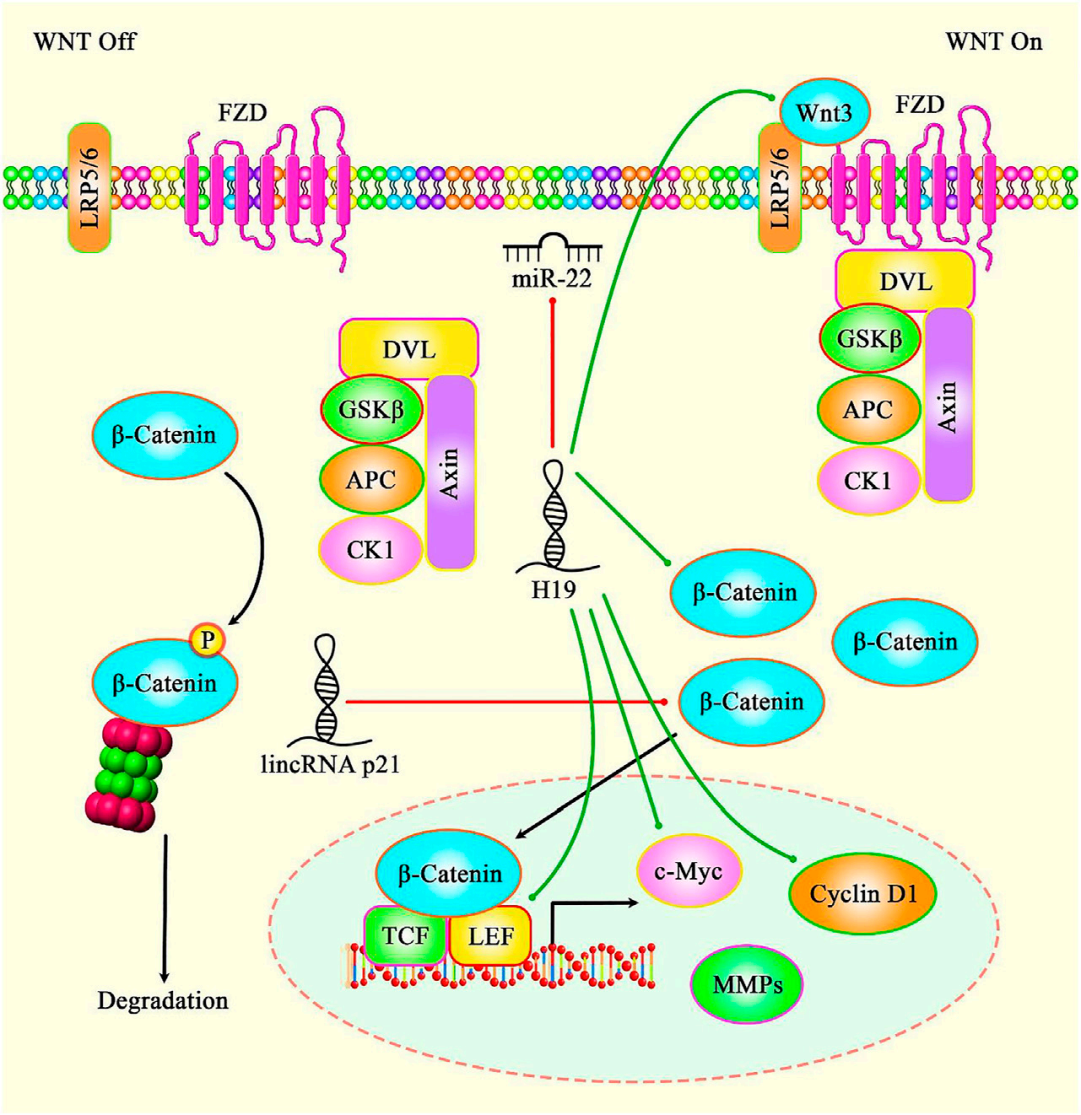
A schematic diagram of the role of several lncRNAs in the modulation of cellular senescence through Wnt/β-catenin signaling pathway. Accumulating evidence has illustrated that various lncRNAs are important regulatory factors in the biological aging. It has been reported that lncRNA H19 via directly targeting miR-22 could promote H2O2-induced deregulation in nucleus pulposus cell senescence, proliferation, and ECM synthesis through Wnt/β-catenin signaling cascade. Thereby, lncRNA H19 could enhance the expression levels of LEF1, c-Myc, and Cyclin D1 in NPCs (Wang et al., 2018b). Moreover, another research has figured out that lncRNA-p21 via downregulating β-catenin expression could regulate cellular senescence in mesenchymal stem cells (Xia et al., 2017). Green lines indicate the positive regulatory effect among lncRNAs and their targets, and red lines depict negative one among them.

H19 is another lncRNA that participates in the process of aging. This lncRNA has been found to be under-expressed in the endothelial cells of aged mice. Similarly, expression of H19 has been down-regulated in atherosclerotic plaques compared with normal carotid artery. Mecahnistically, H19 silencing has resulted in the upregulation of p16 and p21, thus reducing cell proliferation and inducing senescence *in vitro*. Moreover, H19 silencing has suppressed sprouting capacity of aortic rings in young mice. Endothelial-specific H19 deficiency has resulted in elevation of systolic blood pressure and susceptibility to hindlimb ischemia. Exon array analyses have indicated participation of H19 in IL-6 signaling. Moreover, ICAM1 and VCAM1 have been found to be upregulated following H19 silencing. Based on the results of luciferase reporter screening, STAT3 is activated after H19 silencing and suppressed upon H19 up-regulation (Hofmann et al., 2019). In addition to this mechanism, H19 targeting miR-22 has an impact in modulation of H_2_O_2_-induced dysregulation in cellular senescence via Wnt signaling (Figure 2) (Wang et al., 2018a).

HCP5 is another lncRNA that regulates cellular senescence through modulation of expression of iR-128. In fact, HCP5 silencing can induce senescence in glioma cells through this rout, thus enhancing their radiosensitivity (Wang et al., 2021b). In addition to these lncRNAs, a number of long intergenic RNAs (lincRNAs) such as LINC00623, LINC00673, LINC01255, Linc-ASEN and lincRNA p21 have been found to affect cellular senescence. For instance, LINC00623 affects IL-1β-mediated degradation of extracellular matrix, thus being involved in the process of senescence of osteoarthritis chondrocytes through acting as a sponge for miR-101 and influencing expression of HRAS (Lü et al., 2020). The role of LINC00673 in cellular senesence has been well established in the context of lung camcer (Roth et al., 2018). Moreover, LINC01255 has been shown to modulate senesence of mesenchymal stromal cell and proliferation of acute myeloid leukemia cells via inhibition of expression of MCP-1 (Liu et al., 2021a). Supplementary Table S1 summarizes the role of lncRNAs in senescence.

## miRNAs and Senescence

**FIGURE 3:**
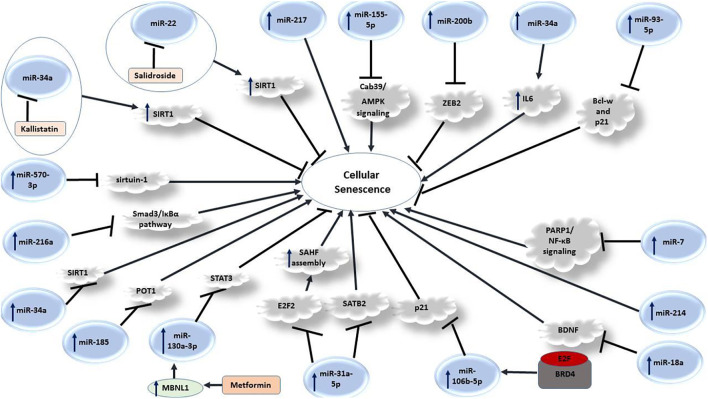
Role of miRNAs in senescence.

Several miRNAs can influence senescence process through different mechanisms. Figure 3 depicts a number of mechanisms through which miRNAs regulate this process.

Let-7g is an example of miRNAs with anti-aging impact on endothelial cells. This miRNA can inhibit expression of LOX-1, a gene that activates aging process. An *in vitro* study in endothelial cells has shown that Ang II can increase expression of aging markers, while decreasing expression of the anti-aging gene SIRT1. Let-7g could exert anti-aging effects on endothelial cells through a LOX-1-independent route (Hsu et al., 2018).

miRNAs can also regulate senescence in the neoplastic conditions. miR-7 is an example of miRNAs being down-regulated in gemcitabine-resistant pancreatic cancer cells. This miRNA can regulate cellular senescence through influencing PARP1/NF-κB pathway. Restoration of miR-7 expression has induced gemcitabine sensitivity in pancreatic cancer cells to. Taken together, miR-7 can regulate cellular senescence and ameliorate resistance to gemcitabine through influencing PARP1/NF-κB pathway (Ye et al., 2021).

Experiments in an animal model of acute myocardial infarction have shown over-expression of miR-18a. Moreover, BDNF has been found to be down-regulated. Suppression of miR-18a expression has led to increase in the expression levels of p-AKT and p-mTOR and the quantities of senescent cells. Moreover, this intervention has reduced levels of Beclin1, LC3-II, p62 decreased and autophagy (Locatelli et al., 2019).

Virus-encoded miRNAs can also affect senescence process in host cells. For instance, EBV-miR-BART3-3p (BART3-3p) has been shown to promote growth of gastric cancer and inhibit the senescence process of these cells. Moreover, BART3-3p inhibits senescence of these cells in a mouse model of gastric cancer and suppress infiltration of NK cells and macrophages in tumors through changing the senescence-associated secretory phenotype. Functionally, BART3-3p targets TP53 and induces down-regulation of p21 (Xu et al., 2019a).

miRNAs can also participate in cytokine-induced premature senescence in endothelium. Expression assay in cellular model of this type of senescence has shown differential expression of eight miRNAs in TNF-α-induced premature senescence cells. Among dysregulated miRNAs have been members of the miR-17-92 cluster. These miRNAs have been known to regulate angiogenesis. hsa-miR-20b participates in this process through targeting RBL1. Silencing of hsa-miR-20b has reduced premature senescence in the TNF-α-treated cells, enhanced their proliferation, elevated RBL1 levels but reduced levels the senescence marker p16INK4a (Wong et al., 2017).

**FIGURE 4:**
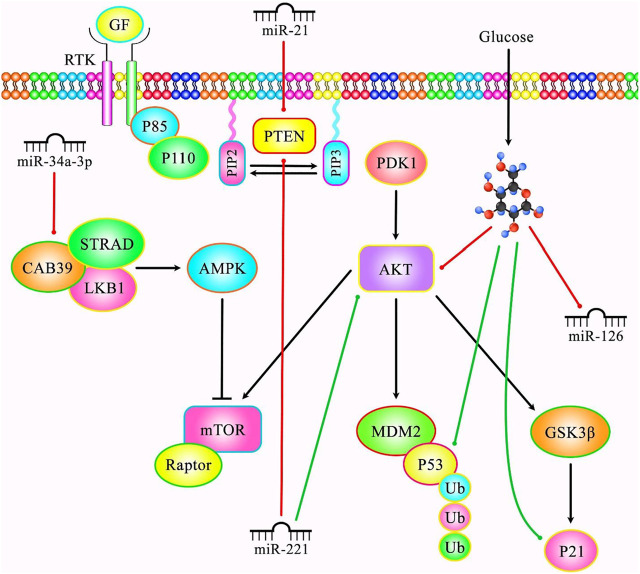
A schematic diagram of the role of several miRNAs in the senescence process through regulating PI3K/AKT/mTOR signaling cascade. Mounting evidence has demonstrated that aberrant expression of various miRNAs could be correlated with cellular senescence. As an illustration, a recent study has detected that miR-221 could play a crucial role in modulating proliferation, chemotherapy sensitivity and senescence in lung cancer cells via downregulating PTEN and upregulating AKT expression levels (Wang et al., 2018c). Moreover, another research has revealed that high glucose could result in premature senescence of human glomerular mesangial cells via reducing miR-126 and p-Akt expression levels and promote in the expression of p53, p21 and Rb proteins in the high-dose d-glucose group (Cao et al., 2019). Furthermore, another study has detected that miR-21 could elevate cardiac aging induced by D-gal and Dox via suppressing PTEN (Bei et al., 2018). In addition, miR-34a-3p could enhance the senescence of dental pulp stem cells via inhibiting CAB39 expression through the AMPK/mTOR signaling pathway (Zhang et al., 2021). Green lines indicate the positive regulatory effect among miRNAs and their targets, and red lines depict negative one among them.

miR-20b is another miRNA that regulates cellular senescence. This miRNA suppresses senescence of endothelial cells via modulating Wnt/β-catenin signals through the TXNIP/NLRP3 axis (Dong et al., 2020). Similarly, delivery of miR-21 and miR-217 by extracellular vesicles can induce cellular senescence in endothelial cells (Mensà et al., 2020). Figure 4 demonstrates the role of various miRNAs in modulating cellular senescence through PI3K/AKT/mTOR signaling pathway.

Supplementary Table S2 summarizes the role of miRNAs in cellular senescence.

## circRNAs and Senescence

**FIGURE 5:**
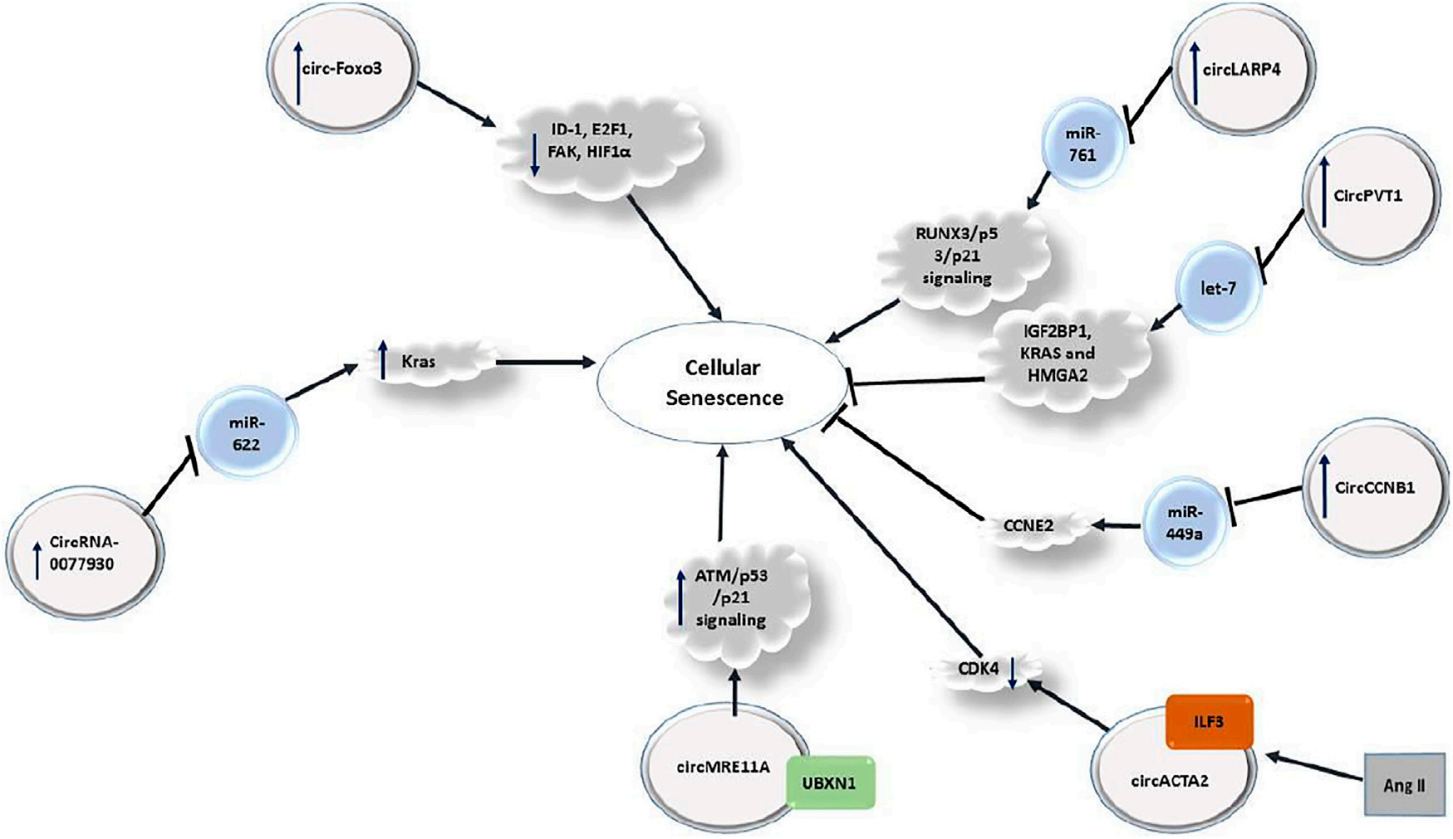
Role of circRNAs in senescence.

Recent investigations have verified contribution of a number of circRNAs in regulation of cellular senescence in different tissues (Figure 5). For instance, circRNA-0077930 secreted from HG-HUVEs-Exos has been shown to increase senescence of VSMCs by reducing miR-622 expression and up-regulation of KRAS, p21, p53 and p16 expression (Wang et al., 2020c). Another study has shown over-expression of circ-Foxo3 in heart tissue of aged humans and mice. Up-regulation of this circRNA has been associated with expression of cellular senescence markers. Forced over-expression of circ-Foxo3 could aggravate doxorubicin-induced cardiomyopathy, while its silencing has relieved this process. Notably, circ-Foxo3 silencing inhibited the senescence process in embryonic fibroblasts of mice. Circ-Foxo3 is able to interact with ID-1, E2F1, FAK, and HIF1α (Du et al., 2017).

Regulation of cellular senescence by circRNAs has also investigated in neoplastic conditions. For instance, in hepatocellular carcinoma cells, up-regulation of circLARP4 could induce cellular senescence and cell cycle arrest through modulation of miR-761/RUNX3 axis (Du et al., 2019).

Comparison of expression profile of circRNAs in proliferating and senescent fibroblasts has shown differential expression of several circRNAs among being circPVT1. This circRNA is produced through circularization of a PVT1 exon. Expression of circPVT1 is decreased in senescent fibroblasts. Mechanistically, down-regulation of circPVT1 in proliferating fibroblasts triggers cellular senescence, enhanced senescence-related β-galactosidase activity, increased CDKN1A/P21 and p53 levels, and decreased cell proliferation. CircPVT1 has been predicted to sequester numerous miRNAs, among them being let-7 which has been enriched following circPVT1 pulldown. Consistent with this interaction, antagonizing endogenous let-7 has induced cell proliferation. CircPVT1 silencing has enhanced cellular senescence and overturned the proliferative effect of let-7. Expression of IGF2BP1, KRAS and HMGA2 have been decreased by circPVT1 silencing (Panda et al., 2017). CircCCNB1 is another circRNA that inhibits cellular senscence via sponging miR-449a and affecting CCNE2 (Du et al., 2019). Moreover, circACTA2 participates cellular senescence through regulation of interaction between ILF3 and CDK4 (Ma et al., 2021).

Table 1 summarizes the role of circRNAs in regulation of cellular senescence.

**TABLE 1:** circRNAs and cellular senescence.

circRNA	Pattern of expression during cellular senescence	Clinical samples/Animal model	Assessed cell lines	Targets/Regulators	Signaling pathways	Description	References
CircRNA-0077930	Upregulated	_	HUVECs and VSMCs	miR-622, KRAS, p21, p53 and p16	_	CircRNA-0077930 from HG-HUVEs-Exos increases senescence of VSMCs by reducing miR-622 expression and up-regulation of Kras, p21, p53 and p16 expression	Wang et al. (2020c)
circ-Foxo3	Upregulated	20 heart samples of aged patients and model of Doxorubicin-induced cardiomyopathy was induced in adult mice	Primary cardiomyocytes isolated from neonatal and 12 weeks heart tissues of strain C57 mice	ID-1, E2F1, FAK, and HIF1α	_	∆ circ-Foxo3: ↓ senescence of mouse embryonic fibroblasts	Du et al. (2017)
circLARP4	Upregulated	70 pairs of HCC tissues and ANCTs/male nude mice	normal human liver cell line QSG-7701 and HCC cell lines Huh7, Hep3B, SMMC7721 and HepG2	miR-761, RUNX3	p53/p21 signaling	↑↑ circLARP4: ↑ senescence and cell cycle arrest and ↓ HCC cell proliferation via miR-761/RUNX3 axis	Du et al. (2019)
circPVT1	Downregulated	_	Human WI-38 fibroblasts, MCF7 breast carcinoma cells, and IMR-90 lung fibroblasts, Human breast epithelial MCF10a cells, lung epithelial BEAS-2B cells, and lung adenocarcinoma A549 cells, Human non-small cell lung carcinoma H1299 cells	let-7, IGF2BP1, KRAS and HMGA2	_	∆ circPVT1: ↑ cell senescence, ↓ cell proliferation	Panda et al. (2017)
circCCNB1	Downregulated	_	Human diploid fibroblasts 2BS and IMR-90 cells, HEK293 T cell lines	miR-449a, CCNE2	_	∆ circCCNB1: ↑ senescence in young 2BS cells and ↓cell proliferation	Du et al. (2019)
circACTA2	Upregulated	12 patients with high blood pressure and 12 patients without high blood pressure	Human aortic smooth muscle cells (VSMCs)	Ang II, ILF3, CDK4	_	∆ circACTA2: ↓ Ang II-induced VSMC senescence	Ma et al. (2021)
						Ang II facilitates the interaction between circACTA2 and ILF3, thus decreases CDK4 mRNA stability and protein expression	
circMRE11A	Upregulated	10 ARC-C, 10 ARC-N, 10 ARC-P patients and 10 controls/ICR mouse	Human LEC line (SRA01/04 cell)	UBXN1	ATM/p53/p21 signaling	circMRE11A could bind to UBXN1 and increase activation of ATM and ATM/p53/p21 signaling, so induces LECs cell-cycle arrest and senescence	Liu et al. (2021c)

(∆, knock-down or deletion; ANCTs, adjacent non-cancerous tissues)

## Discussion

The data presented above indicated contribution of miRNAs, lncRNAs and circRNAs in the modulation of senescence. The effective role of some lncRNAs such as ANRIL on cellular senescence has been verified in both malignant and non-malignant conditions. LncRNAs can affect senescence through sponging miRNAs. For instance, ANRIL/miR-181a, GAS5/miR-223, GAS5/miR-665, H19/miR-22, HCP5/miR-128, HEIH/miR-3619-5p, LINC00623/miR-101, MALAT1/miR-92a-3p, MEG3/miR-16-5p, MEG3/miR-128, MIAT/miR-22-3p, MIAT/miR-302, NEAT1/miR-221-3p, RP11-670E13.6/miR-663a and TRPC7-AS1/miR-4769-5p are examples of lncRNAs/miRNAs that corporately participate in the senescence. Similarly, there are several examples of circRNAs that participate in this process through sponging miRNAs. CircRNA-0077930/miR-622, circLARP4/miR-761, circPVT1/let-7 and circCCNB1/miR-449a are few uncovered circRNAs/miRNAs axes functioning in the regulation of senescence. Thus, it is obvious that lncRNAs, circRNAs and miRNAs work in a complex network to influence cellular senescence.

PI3K/AKT, Wnt/β-catenin, AMPK/mTOR/ULK1, MAPK, p53/p21, p16/pRb, NF-κB/TNF-α, RAF/MEK/ERK, JNK, Smad3/IκBα and Smad3/NF-κB signaling pathways are among pathways, which are regulated by non-coding RNAs in the process of cellular senescence.

Several non-coding RNAs that regulate cellular senescence interact with p53/p21 and/or phosphorylation of pRb/p16 pathways. These two interconnected and partly exclusive processes participate in all terminating events of senescence irrespective of varied initiating factors (Cai et al., 2021). Stress-dependent up-regulation of p53 induces PTEN, which in turn suppresses two p53 inhibitors, namely, PI3K/AKT and SIRT1/FOXO3a signals (Hekmatimoghaddam et al., 2017). Thus, interaction of non-coding RNAs with these pathways is another route of participation of non-coding RNAs in the cellular senescence.

Induction of cellular senescence can enhance response of cancer cells to therapeutic options. Thus, this strategy can be used in combination with conventional anti-cancer therapies. Identification of the impact of non-coding RNAs in the regulation of cellular senescence has a practical significance in cancer treatment. Moreover, since abnormal expression of non-coding RNAs in cancer cells can have anti-senescence effects, evaluation of levels of these transcripts in tumor samples can facilitate prior evaluation of response of tumor cells to chemoradiotherapy.

Manipulation of senescence process has been suggested as a therapeutic option for diverse disorders. While pro-senescent treatments are desirable therapies in malignant conditions and for continuing tissue repair processes, anti-senescent treatments can be helpful for removal of senescent cells in the contexts of ageing or chronic injuries (Muñoz-Espín and Serrano, 2014).

Taken together, non-coding RNAs have essential roles in the modulation of cellular senescence and pathoetiology of senescence-related disorders. This field represents an unexplored research area for design of novel therapies for a wide variety of human disorders, including both aging-related disorders and cancers.
