# Supplementary material for: Emerging Role of Non-Coding RNAs in Senescence
Source: Front Cell Dev Biol. 2022 Jul 5;10:869011. doi: 10.3389/fcell.2022.869011 (PMC9294638; doi:10.3389/fcell.2022.869011)
Supplement: Supplementary file 1 [file DataSheet1.docx]

Supplementary Material

**Supplementary Table 1**. **LncRNAs and cellular senescence** (HCC: hepatocellular carcinoma, ANCTs: adjacent non-cancerous tissues, ∆: knock-down or deletion, NPCs: nucleus pulposus cells, ECM: extracellular matrix, MIF: macrophage migration inhibitory factor, DOX: chemotherapy drug doxorubicin, MSCs: mesenchymal stem cells, HA-VSMCs: human aortic vascular smooth muscle cells, IDD: Intervertebral disc degeneration, OA: osteoarthritis, ECM: extracellular matrix, MSCs: mesenchymal stem cells, CSM: cigarette smoke-media suspension, AECII: type II alveolar epithelial cell, CRC: colorectal cancer, PTB: Preterm birth).

| lncRNA | Pattern of Expression during cellular senescence | Clinical Samples/Animal Model | Assessed Cell Lines | Targets / Regulators | Signaling Pathways | Description | Reference |
| --- | --- | --- | --- | --- | --- | --- | --- |
| ANRIL (CDKN2B-AS1) | downregulated | _ | vascular smooth muscle cells (VSMCs) | miR-181a, Sirt1 | _ | LncRNA-ANRIL reduced cell senescence by regulating miR-181a/Sirt1-AS. | (10) |
|  | downregulated | female BALB/c nude mice | human cervical normal cell line Etc1/E6E7 and cancer cell lines (HeLa, C4‐1, SiHa, Ca Ski cells) | miR-181a-5p, TGFβI | _ | ∆ CDKN2B-AS1: ↓ proliferation, invasion, migration and ↑ apoptosis and senescence in cervical cancer via miR-181a-5p/TGFβI axis | (11) |
| GAS5 | downregulated | _ | Endothelial progenitor cells (EPCs) | miR-223, NAMPT | PI3K/AKT signaling | ∆ GAS5: ↓ EPC proliferation and ↑ senescence via miR-223/NAMPT axis | (12) |
|  | downregulated | _ | Human aorta VMSCs | miR-665, SDC1 | _ | ↑↑ GAS5: ↓ miR-665 level and VSMC senescence | (13) |
|  | upregulated | male C57BL/6 mice | HT22 cell line | _ | _ | GAS5 and its interacting proteins are involved in senescence and brain aging. | (14) |
| Gm14230 | downregulated | male C57BL/6N mice | Hepa1-6, NIH3T3 and ONS-76 | Ezh2, Tgif2 | _ | ∆ Gm14230: ↓ cell growth and ↑ cellular senescence | (15) |
| GUARDIN | downregulated | _ | HepG2, A549, H1299, 293T, and HAFF | FOSL2, LRP130-PGC1α, FOXO4 –p21 | _ | GUARDIN acts to facilitate interactions between LRP130 and PGC1α so represses expression of FOXO4 and its target p21. Therefor inhibits cellular senescence.  Rapamycin inhibits FOSL2 and subsequently increased GUARDIN expression. | (16) |
| H19 | downregulated | Cdh5(PAC)-CreERT2-H19 flox mice on a C57Bl6/J background | Human umbilical vein endothelial cells (HUVECs), HeLa, Hek293T | STAT3, p16, p21 | _ | ∆ H19: ↑ p16, p21, and senescence, ↓ proliferation in vitro  ∆ H19: ↓ sprouting capacity in vivo | (17) |
|  | upregulated | _ | nucleus pulposus cells (NPCs) | miR-22, LEF1 | Wnt/β-catenin signaling | H19 could increase H2 O2 -induced degenerative changes by boosting cell senescence, elevating levels of ADAMTS-5 and MMPs protein and Collagen I content, as well as decreasing NPC proliferation through activating Wnt/β-catenin signaling | (18) |
| HCP5 | downregulated | 20 glioma tissues and 5 Human normal brain tissues | Glioma cell lines (A172, U87, U251, SHG44) | miR-128 | _ | ∆ HCP5: ↓ proliferation and ↑ radiosensitivity in glioma cells by intervening in cellular senescence | (19) |
| HEIH | downregulated | 25 Ovarian cancer tissues and 25 corresponding non- tumor tissues | human ovarian cancer cell lines (OVCA429, OVCA433, and OVCAR3) and normal human ovarian epithelial cells (IOSE144) | miR-3619-5p/CTTNBP2 | _ | HEIH increased proliferation, migration, and invasion, whereas decreased cell senescence in ovarian cancer via miR-3619-5p/CTTNBP2 axis. | (23) |
| HOTAIR | upregulated | rats | nucleus pulposus (NP) cells | _ | AMPK/mTOR/ULK1 pathways | ↑↑ HOTAIR: ↑ autophagy to promote NP cell apoptosis, senescence, and ECM catabolism  ∆ HOTAIR: ↓ IDD in rats | (24) |
|  | _ | _ | Bone marrow derived MSCs | _ | _ | HOTAIR has an impact on differentiation of MSCs, thus impacts senescence-associated DNAm. | (25) |
| LINC00623 | downregulated | normal cartilage tissues and degenerated cartilage tissues | primary OA chondrocytes | miR-101, HRAS | MAPK signaling | ∆ LINC00623: ↑ IL-1β-induced OA chondrocyte apoptosis, senescence, and ECM degradation via miR-101, HRAS axis | (20) |
| LINC00673 | downregulated | _ | A549 cells and IMR-90 cells | p53 | _ | ∆ LINC00673: ↑ cellular senescence  ↑↑ LINC00673: ↓ p53 translation | (21) |
| LINC01255 | _ | _ | human mesenchymal stromal and acute myeloid leukemia cells | MCP-1, BMI1 | p53-p21 pathway | LINC01255 regulates the senescence of human mesenchymal stromal and the proliferation of acute myeloid leukemia cell by suppressing the transcription of MCP-1. | (22) |
| Linc-ASEN | downregulated | female BALB/c nude mice | MCF7, HEK293T, SW620, HDFs, H1299, MCA10A | UPF1, p21, PRC1 and PRC2 | _ | Linc-ASEN reducing cellular senescence by inhibiting p21 transcription. | (26) |
| lincRNA‑p21 | upregulated | _ | bone marrow‑derived MSCs from male C57BL/6 mice | _ | Wnt/β‑catenin signaling pathway | ∆ lincRNA‑p21: ↓ MSC senescence by interacting with the Wnt/β‑catenin signaling pathway | (27) |
|  | upregulated | _ | WST-1, HL-1 | _ | Wnt/β‑catenin signaling pathway | ∆ lincRNA‑p21: ↑ proliferation and cellular viability, ↓ Dox‑induced cardiac senescence | (28) |
| lnc‑HRK‑2:1 | upregulated | _ | NP cell | CCL5 and PNPT1 | _ | High levels of lnc‑HRK‑2:1 prompts nucleus pulposus cell senescence in intervertebral disc degeneration. | (29) |
| lncRNA-ES3 | upregulated | _ | HA-VSMCs | miR-95-5p, miR-6776-5p, miR-3620-5p, and miR-4747-5p, Bhlhe40 | _ | ↑↑ lncRNA-ES3: ↑ calcification/senescence of HA-VSMCs | (30) |
|  | upregulated | _ | HA-VSMCs | miR-34c-5p, BMF | _ | ∆ lncRNA-ES3: ↓ calcification/senescence of HA-VSMCs by suppressing miR-34c-5p | (31) |
| lncRNA-OIS1 | _ | _ | BJ/ET/RasV12, TIG3/ET/RASV12, Ecopack 2 and HEK293-T cells | DPP4 | _ | ∆ lncRNA-OIS1: ↑ bypass of senescence, proliferation rate, expression of cell-cycle-associated genes, ↓ abundance of the cell-cycle inhibitor CDKN1A  LncRNA-OIS1 modulates senescence induced by RAS by regulating DPP4 activation. | (32) |
| lncRNA-SAL1 | downregulated | human lung tissues from patients | type II alveolar epithelial cell (AECII) | SIRT1 | _ | lncRNA-SAL1-mediated SIRT1 signaling decreased senescence of CSM-induced AECIIs. | (33) |
| MALAT1 | downregulated | _ | Human-induced pluripotent stem cell–derived cardiomyocytes, Human adipose–derived MSCs | miR-92a-3p, ATG4a | _ | ∆ MALAT1 in MSCs: ↓ rejuvenation induced by exosome  MALAT1/miR-92a-3p/ATG4a could suppress Dox-induced senescence. | (34) |
|  | downregulated | C57BL/6 male mice | Human umbilical mesenchymal stem cells (UMSC), rat cardiomyocyte cell line H9C2 | _ | NF-κB/TNF-α signaling pathway | UMSC-derived exosomes reduced aging-induced cardiac dysfunction through releasing MALAT1 that in turn inhibits the NF-κB/TNF-α signaling pathway. | (35) |
|  | downregulated | 48 patients with GBC and 16 patients with cholecystitis as controls | GBC cell lines, GBC-SD, SGC-996, NOZ and OCUG1 | ABI3BP, EZH2 | _ | ∆ MALAT1: ↑ cell senescence and ↓cell growth in gallbladder cancer (GBC) | (36) |
| MAYA | upregulated | HFD-fed Golden hamsters | human immortalized normal hepatocyte cell line LO2 cells | YAP | _ | ∆ MAYA: ↓ iron overload and cellular senescence via promoting YAP in PA-treated hepatocytes. | (37) |
| MEG3 | upregulated | _ | A549 and MCF-7 cells | miR-16-5p, VGLL4 | _ | MEG3 increases cell senescence via the miR-16-5p/VGLL4 axis. | (38) |
|  | downregulated | C57BL/6J mice | Human Umbilical Vein Cells (HUVECs) | CDKN2A | _ | ∆ MEG3: ↑ cellular senescence of endothelial cells in obesity, so impairs glucose homeostasis and insulin signaling | (39) |
| MEG3 | downregulated | umbilical vein of healthy young (aged 1–10 yr) and older (aged 70–80 yr) volunteers/ C57BL/6 female mice | human umbilical vein endothelial cells (HUVECs) | miR-128, Girdin | _ | MEG3 promotes platelet phagocytosis, therefore protecting VECs from senescence via miR-128 and Girdin axis. | (40) |
| MIAT | downregulated | 20 pairs of HCC tissues and ANCTs | 293T, HepG2, SMMC-7721, PLC/PRF/5, Huh7 and SK-hep-1, 2BS, IMR90 and MRC-5 | miR-22-3p, sirt1, SASP | p53/p21 and p16/pRb pathways | ∆ MIAT: ↑ cellular senescence, and ↓ HCC progression, ↓ proliferation, and activated the tumor suppressor pathway (p53/p21 and p16/pRb), ↑ SASP levels | (41) |
|  | downregulated | _ | MCF7 cell line | mir-302, mir-150, mir-29c, p16 and Cox2 | _ | ∆ MIAT: ↓ proliferation, ↑ senescence and G1 arrest in MCF7 cell line in breast cancer | (42) |
| MIR31HG | upregulated | TCGA database | human diploid cell lines TIG3 and BJ, HEK293T cells | SASP components, YBX1 | _ | MIR31HG has a dual role in senescence depending on its localization via interaction with p16/CDKN2A or interacting with YBX1 and regulating SASP. | (43) |
| NEAT1 | _ | Male C57/Bl6 mice | BM‑MSCs | miR-221-3p, Sirt2 | _ | ∆ NEAT1: ↓ exosomeMIF-induced anti-senescent effect against Dox ExosomeMIF treatment: ↓ Dox-related cardiac injury through inhibiting miR-221-3p (a target of NEAT1) | (44) |
| OVAAL | downregulated | 33pairs of colon cancer tissues and ANCTs | ME4405, TRAIL.S, UMI-77.S, HCT116, P493-6, and HAFF cells | p27, c-Myc, STK3 and Raf-1 | RAF/MEK/ERK signaling | ∆ OVAAL: ↑ cellular senescence | (45) |
| PANDA | downregulated | 48 HCC patients following liver transplantation and 84 HCC patients following liver resection/ immunodeficient mice | Hepatocellular carcinoma cell lines, HCC LM3 and Huh7 | _ | _ | PANDA suppresses transcriptional activity of senescence associated inflammatory factor IL8, that leads to inhibition of cellular senescence in hepatocellular carcinoma. | (46) |
| PLK4 | upregulated | male BALB/c nude mice | HepG2 | YAP | _ | Talazoparib-induced PLK4 via YAP inactivation promotes cellular senescence to inhibit liver cancer cell viability and growth. | (47) |
| RP11-670E13.6 | _ | _ | 293T cells | miR-663a, CDK4, CDK6, hnRNPH | _ | RP11-670E13.6 delayed cellular senescence during UV irradiation-induced skin photoaging.  ∆ RP11-670E13.6: ↑ cellular senescence, ↑ DNA damage  ↑↑ MiR-663a: ↑ cellular senescence by targeting CDK4 and CDK6  hnRNPH inhibited RP11-670E13.6 expression. | (48) |
| RPPH1 RNA | upregulated | _ | Human fibroblasts WI-38 and IDH4 | AUF1 | _ | RPPH1 RNA accumulates in mitochondria of senescent fibroblasts and modulated by AUF1. | (49) |
| SENEBLOC  (SBLC) | _ | male nude mice | HCT116, A549, IMR90, HAFF, 293T and P493-6 cells | p53, MDM2, p21, miR-3175, HDAC5 | _ | SENEBLOC facilitates the association of p53 with MDM2 (to promote p53 degradation) in order to decrease p21 transactivation besides SENEBLOC regulates HDAC5 expression (by sponging miR-3175) to silence p21 gene promoter so it could decrease cellular senescence through two mechanisms.  c-Myc increased SBLC levels. | (50) |
| SNHG29 | upregulated | 10 placentas of women who had PTB with labor (n, 8 PTB with no labor, 10 full- term birth with labor, 10 full-term birth with no labor | HTR8/SVneo cells | p53/p21,  β-galactosidase | _ | High levels of SNHG29 were observed in placentas from women who delivered preterm with labor which leads to senescence in vivo. | (51) |
| SNHG6 | downregulated | Oncomine database (69 gastric tissue samples) and 114 GC patients and 99 healthy controls/  male BALB/c nude mice | GES-1, MKN45, BGC-823, MGC-803, SGC-7901, AGS, and 293T | p21, EZH2 | JNK pathway | ∆ SNHG6: ↓ proliferation, and ↑ cellular senescence in gastric cancer (GC) | (52) |
| TRMP-S | downregulated | _ | H1299, 293T, HCT116, A549, SKOV3, Mel-RM, U2OS, NCM460, HepG2, and HAFFs | FUBP3, p53, RPL26,p27, UHRF1 | _ | TRMP-S stabilizes UHRF1 protein levels (an inhibitor of p27)  TRMP-S and FUBP3 Interactions prevent p53 mRNA interactions with RPL26 ribosomal protein so prevents p53 translation and limits p27 expression.  TRMP-S inhibits cellular senescence. | (53) |
| TRPC7-AS1 | Upregulation of TRPC7-AS1 increased NPC senescence. | 18 normal lumbar disc and 26 degenerative specimens | NPC cells, 293T | miR-4769-5p, HPN | _ | ↑↑ miR-4769-5p (a target of TRPC7-AS1): ↓ HPN expression, ↓ NPC senescence, ↑ NPC viability, and ↑ ECM synthesis | (54) |
| USP2-AS1 | downregulated | male athymic nude mice | A549, HCT116, IMR90, and HEK293T cell lines | E2F1, c-Myc | _ | c-Myc-activated USP2-AS1 suppresses cellular senescence and plays the role of oncogenic molecule by inducing E2F1 expression. | (55) |
| VIM-AS1 | downregulated | 35 pairs of CRC tissues and ANCTs | SW1116, SW480, HT29 and SW48, colon cancer cell lines | _ | _ | ∆ VIM-AS1: ↓  proliferation and ↑ apoptosis, cellular senescence and cell cycle arrest | (56) |

**Supplementary Table 2**. **microRNAs and cellular senescence** (∆: knock-down or deletion, COPD: Chronic obstructive pulmonary disease, YMSCs: young human mesenchymal stem cells, AMSCs: aged human mesenchymal stem cells, COPD: chronic obstructive pulmonary disease, VSMCs: vascular smooth muscle cells, SASP: senescence-associated secretory phenotype, VC: vascular calcification, AMI: acute myocardial infarction, BMSCs: bone marrow stromal cells, SAHF: senescence-associated heterochromatin foci, CAD: coronary artery disease, VC: Vascular calcification, SMC: vascular smooth muscle cell, AVN A: Avenanthramide A, HSCs: hepatic stellate cells, NFs: normal fibroblasts, CAFs: Cancer-associated fibroblasts, Yan: Yangonin, OMST: Olmesartan, BLM: bleomycin, HCC: hepatocellular carcinoma, HCV: Hepatitis C virus, hMSCs: human mesenchymal stem cells, T2D: type 2 diabetes, OA: osteoarthritis, PG: proteoglycan, ANCTs: adjacent non-cancerous tissues).

| microRNA | Pattern of Expression during cellular senescence | Clinical Samples/Animal Model | Assessed Cell Lines | Targets / Regulators | Signaling Pathways | Description | Reference |
| --- | --- | --- | --- | --- | --- | --- | --- |
| let-7g | downregulated | _ | Human umbilical vein endothelial cells (HUVECs) | _ | _ | MicroRNA let-7g reduced angiotensin II-induced endothelial senescence regardless of the presence or absence of LOX-1. | (58) |
| EBV-miR-BART3-3p (BART3-3p) | downregulated | 20 EBVaGC and 20 non-EBVaGC patient/  nude mice | SGC7901 (EBV-negative GC cell line) and KATOIII (GC cell line) cells, AGS (EBV-negative GC cell line) cells, AGS-EBV (EBV-positive GC cell line), HEK293 cells | TP53 and p21 | _ | ↑↑ BART3-3p: ↓ senescence of gastric cancer cells and ↓ TP53 and p21 | (61) |
| miR-7 | upregulated | 100 pancreatic tumor tissues and 80 adjacent non-tumor tissues | human pancreatic ductal adenocarcinoma PANC-1 cell line | _ | PARP1/NF-κB signaling | miR-7 promotes cellular senescence and reduces gemcitabine resistance by targeting the PARP1/NF-κB axis in pancreatic cancer cells. | (59) |
| miR-18a | upregulated | specific pathogen free (SPF) Wistar rats | Rat cardiomyocytes H9c2 cells, 293T cells | BDNF | _ | ∆ miR-18a: ↓ hypoxia-induced H9c2 cell senescence and ↑ hypoxia-induced H9c2 cell autophagy by up-regulating BDNF expression in AMI | (60) |
| hsa-miR-20b | upregulated | _ | human pulmonary microvascular endothelial cells (HMVEC-Ls) | RBL1 | _ | ∆ hsa-miR-20b: ↓ premature senescence in the TNF-α-treated HMVEC-Ls, protein expression of p16INK4a and ↑ cell proliferation, and RBL1 mRNA expression level | (62) |
| miR‑20b | downregulated | _ | Human umbilical vein endothelial cells (HUVECs) | TXNIP | Wnt/β‑catenin pathway | ↑↑ miR‑20b: ↓ H2O2‑induced cell senescence, ↑ cell viability via targeting TXNIP | (63) |
| miR-21 and miR-217 | upregulated | _ | on-senescent (control) and senescent (SEN) human umbilical vein endothelial cells (HUVECs) and HAECs | DNMT1, SIRT1 | _ | miR-21 and miR-217 delivered from small extracellular vesicles paly a roles as pro-senescence effectors to endothelial cells via targeting DNMT1 and SIRT1. | (64) |
| miR-21-5p/203a-3p | upregulated | hyperlipidemic rats | HUVECs | Drp1 | _ | ↑↑ miR-21-5p/203a-3p: ↑ox-LDL-induced endothelial senescence via targeting Drp1 directly or indirectly | (65) |
| miR-21 | upregulated | male C57BL/6 mice and d-gal-induced pseudo-aging mouse model | Neonatal rat cardiomyocytes (NRCMs) | PTEN | _ | ↑↑ miR-21: ↑ d-gal-induced cardiac alterations and Dox-induced cardiomyocyte senescence via targeting PTEN | (66) |
| miR-22 | upregulated | _ | 2BS cell line from human fetal lung fibroblasts | SIRT1 | _ | Salidroside inhibits cellular senescence by reducing miR-22 and increasing the levels of SIRT1. | (67) |
| miR-23a | upregulated | 104 CAD patients and 50 controls | Peripheral blood mononuclear cells (PBMCs) from patients and controls | TRF2 | _ | ↑↑ miR-23a: ↑ telomere shortening and cellular senescence in coronary artery disease (CAD) via targeting TRF2 | (68) |
| miR-29a and miR-30c | _ | _ | hMSCs from the adipose tissues of three donors | SOD2, DNMT3A | _ | miR-29a and miR-30c restore SOD2 expression by repressing DNMT3A expression, consequently alleviate oxidative stress in hMSCs and rescue proliferation defects, mitochondrial dysfunction, and premature senescence. | (69) |
| miR-29 | upregulated | _ | Primary mouse embryonic fibroblasts (MEFs) from embryos of C57BL/6 mice, human embryonic fibroblasts (HEFs) from human female embryos, primary human umbilical vascular endothelial cells (HUVECs), HEK293T cells | _ | TGF-β signaling | Loss of H4K20me3 induced by the senescent TGF-β/miR-29 pathway leads to cardiac aging in vivo. | (70) |
| miR-30c-1 | downregulated | _ | hCECs | _ | _ | ↑↑ miR-30c-1: ↑ proliferation of hCECs by ameliorating the TGF- β1-induced senescence of hCECs and decreasing cell death of hCECs | (71) |
| miR-30 | downregulated | _ | BJ cells, WI38, U2OS-HR reporter cells, 293T and LinX-A cells | CHD7 and TNRC6A | p16 INK4A and p53 pathways | ↑↑ miR-30: ↓ senescence and ↑ cancer by targeting CHD7 and TNRC6A | (70) |
| miR‑30a | upregulated | _ | vascular smooth muscle cells (VSMCs) | Beclin1 | _ | Rapamycin treatment: ↓ miR‑30a expression, and senescence in VSMCs, and ↑ Beclin1 expression in both young and aging cells, and autophagy in VSMCs  miR‑30a reduced autophagy and increased senescence of VSMCs via targeting Beclin1. | (72) |
| miR-31a-5p | upregulated | female Sprague‐Dawley rats | BMSCs from young rats, middle‐aged rats and aged rats , BMSCs from female humans, Bone marrow (BM) cells from 4‐week‐old SD rats, 293T cells | SATB2,E2F2, SAHF | _ | ∆ miR-31a-5p: ↓ differentiation and function of osteoclasts, bone loss and osteoclastic activity of aged rats  Levels of miR-31a-5p were higher in BMSCs-derived exosomes from aged rats compared to those from young rats, and promote osteoclastogenesis and bone resorption.  miR‐31a‐5p contributes to decrease osteogenic differentiation by blocking SATB2 pathway.  miR-31a-5p promotes cellular aging  and SAHF assembly by blocking E2F2 activity. | (73) |
| miR-33-5p | upregulated | _ | Human VECs | ABCA1, CS | _ | Treatment with ox-LDL: ↓ ABCA1, CS levels, and cholesterol efflux, ↑ miR-33-5p expression, apoptosis, aging, and inflammation in dose-dependent manners  ∆ miR-33-5p: ↓ ox-LDL-induced changes in VECs | (74) |
| miR-34a-5p | upregulated | male C57/Bl6 mice | HL-1 murine cardiomyocytes | PNUTS | _ | Upregulation of miR-34a-5p is induced by exosomes derived from PD-1 inhibitor-treated macrophages in cardiomyocytes, and increases cardiac senescence, thus causes cardiac injury in mouse hearts via targeting PNUTS. | (75) |
|  | upregulated | _ | _ | ATM and p21 | _ | p21 is found to be the main effector of a senescence/apoptosis switch that is regulated by ATM/miR-34a-5p axis in non-small cell lung cancer (NSCLC) cells. | (76) |
| miR-34a-3p | upregulated | Human dental pulp tissues from 12 healthy donors | dental pulp stem cells (DPSCs) | CAB39 | AMPK/mTOR Signaling Pathway | ↑↑ miR-34a-3p: ↑ senescent of DPSCs  Metformin treatment: ↓ miR-34a-3p expression and ↑ CAB39 and AMPK/mTOR signaling pathway. | (77) |
| miR-34a | upregulated | female Fisher 344 rats | rat cardiac cells exposed to doxorubicin (DOXO) | Bcl-2 and SIRT1 | _ | ∆ miR-34a: ↓ DOXO-induced cardiac dysfunction represented by myocardial apoptosis, senescence, fibrosis and inflammation by increasing Bcl-2 and SIRT1 | (78) |
|  | upregulated | streptozotocin (STZ)-induced diabetic mice, and Caenorhabditis elegans | HEK293T cells, endothelial progenitor cells (EPCs) | SIRT1 | _ | Kallistatin inhibits cellular senescence by reducing miR-34a and increasing the levels of SIRT1. | (79) |
| miR-34a | _ | 3 llsteatohepatitis patients and 3 healthy controls/ ethanol-fed mice/ male C3H/HeOu/J mice | mouse HSCs and Hepatocytes | transforming growth factor-β1, Smad2, and Smad3, p16, p21, CCL2,  PAI-1, and SIRT1 | _ | Downregulation of miR-34a represses the expression of fibrosis genes in cultured HHSCs and hepatocytes and increases the expression of senescence markers in HHSCs. | (80) |
|  | upregulated | 128 healthy subjects/ JAX™ C57BL/6J mice/ male C57BL/6J mice | human aortic smooth muscle cells (HASMCs) | IL6 | _ | Conditioned medium of miR-34a-overexpressing cells show increased HASMCs senescence and calcification, thus the vascular age-associated miR-34a accelerates  VSMCs SASP activation and plays an role in arterial inflammation and dysfunctions such as VC. | (81) |
|  | upregulated | _ | Human adipose derived mesenchymal stem cells (hAD-MSCs) | SIRT1 | _ | ∆ miR-34a: ↓ cellular senescence, and ↑ proliferation, adipogenic and osteogenic differentiation potency | (82) |
|  | upregulated | JAX C57BL/6J mice | HASMCs | Axl and SIRT1 | _ | miR-34a induces VC via vascular SMC mineralization by reducing cell proliferation and increasing senescence via targeting Axl and SIRT1 | (83) |
|  | upregulated | male Sprague Dawley (SD) rats | Rat embryonic H9c2 myoblasts, BM-MSCs from the femur and tibia of SD rats, | SIRT1 | _ | MSCs show anti-senescence effect by inhibiting miR-34a and increasing the levels of SIRT1. | (84) |
|  | upregulated | _ | Human Retinal Endothelial cells (HuREC) | TrxR2 | _ | ↑↑ miR-34a: ↑ cellular senescence, mitochondrial dysfunction and Loss of antioxidant activities via targeting TrxR2 | (85) |
|  | upregulated | _ | human umbilical vein endothelial cells(HUVECs) | SIRT1 | p53 signal pathway | Notoginsenoside R₁ delays H₂O₂-induced vascular endothelial cell senescence via miR-34a/SIRT1/p53 signal pathway. | (86) |
|  | upregulated | 14 idiopathic pulmonary fibrosis (IPF) lung tissues and 10 normal lung tissues plus 12 IPF lung fibroblasts and 14 age-matched normal controls /  C57BL/6 mice | Human lung fibroblast line MRC-5 | _ | _ | ↑↑ miR-34a: ↑ senescent phenotype in lung fibroblasts and ↓ proliferation | (87) |
| miR-34a | upregulated | 30 T2D patients and 30 controls | MIN6 cells | _ | _ | Levels of miR-34a and oxidative stress marker levels were increased in the middle-aged Indians with T2Din comparison to the controls. | (88) |
|  | upregulated | C57/BL6 mice | Human umbilical vein endothelial cells (HUVECs), mouse lung endothelial cells and HEK293T cells | Let-7g, SIRT1, eNOS | _ | ↑↑ Kallistatin: ↑ levels of Let-7g, SIRT1, eNOS and ↓ levels of miR-34a and endothelial senescence | (89) |
|  | upregulated | _ | H9C2 cells | PNUTS | _ | ↑↑ miR-34a: ↑ DOX-induced H9C2 cell senescence by targeting PNUTS  Extracellular vesicles inhibit H9C2 cell senescence via the miR-34a/PNUTS axis. | (90) |
|  | upregulated | _ | Human non-small cell lung cancer (NSCLC) cell lines A549 and H460 | c-Myc | _ | ↑↑ miR-34a: ↑ IR-induced senescence in NSCLC cells via targeting Myc | (91) |
|  | upregulated | _ | human umbilical vein endothelial cells (HUVEC) | SIRT1 | _ | Total flavonoids decrease miR-34a expression and increase the expression of SIRT1, thus inhibits HUVEC senescence | (92) |
|  | upregulated | _ | primary MSCs of healthy, young and old male Wistar rats | Nampt | NAD +-Sirt1 pathway | ↑↑ miR-34a: ↑ senescence-associated characteristics in young MSCs | (93) |
|  | upregulated | 7 hip OA patients, 7 Knee OA patients, and 8 patients with collum femoris fractures as controls | Human chondrocytes (HC) | DLL1 | PI3K/AKT pathway | ↑↑ miR-34a: ↑ cell death and senescence, ↓ proliferation, and scratch assay wound closure | (94) |
|  | upregulated | C57BL/6 wild-type (WT) and miR-34a−/− mice | Human lung epithelial cell lines A549 and BEAS-2B | SIRT1,E2F3 and cyclin E2 | _ | ↑↑ miR-34a: ↑ cellular senescence and apoptosis and mitochondrial aberrations, thus induces dysfunctions in alveolar epithelial cells (AECs) | (95) |
| miR-34a | upregulated | male Long Evans rats | human hepatocytes | SIRT1 | _ | ↑↑ miR-34a: ↑ hepatocyte senescence and apoptosis via targeting SIRT1 | (96) |
|  | upregulated | 39 pairs of HNSCC tissues and ANCTs | UM-SCC-23, Fadu cell line  and HEK-293T embryonic kidney cell line | FUT1, AXL, and MAP2K1 | _ | ↑↑ miR-34a: ↓ HNSCC growth through inducing cell cycle arrest and senescence via targeting proliferation-associated genes | (97) |
|  | upregulated | _ | hBM-MSCs and U87 cells | SIRT1 | _ | ↑↑ miR‑34a: ↑ DNA damage, ↓ telomere length, telomerase activity and ↓ proliferation of glioma cells | (98) |
| miR-34c-5p | upregulated | 13 better risk,13 intermediate-risk, and 14 poor-risk AML patients and 7 CB,6 normal volunteer BM samples | kG-1a, THP-I and KASUMI-1 cell lines | _ | p53-p21Cip1-Cyclin-dependent kinase (CDK)/Cyclin or p53-independent CDK/Cyclin pathways | ↑↑ miR-34c-5p: ↑ LSCs senescence, and eradication of LSCs in immune deficient mice, and ↓ leukemia development | (99) |
| miR-93-5p | downregulated | _ | Human U251 glioblastoma and H460 lung carcinoma cells | Bcl-w and p21 | _ | miR-93-5p by directly targeting Bcl-w and p21 inhibits cellular senescence. | (100) |
| miR-106b-5p | downregulated | 52 gastric cancer samples and 52 paired normal gastric mucosa samples | Human gastric cancer cell lines AGS, AZ521, NCI-N87, MKN28, MNK45, SGC-7901, SNU-5, SNU-16/ HEK293T cells and MKN28 cells | BRD4, E2F, p21 | _ | ∆ miR-106b-5p: ↑ p21 levels and cellular senescence | (101) |
| miR-124 | upregulated | C57BL/6 mice | primary MEF cells from C57BL/6 mouse, HK293T (a SV40‐transformed embryonic kidney cell line), NIH/3T3 (an immortalized mouse embryonic fibroblasts) | Ccna2, p53 | _ | p53 responsive miR-124 and miR-29 were significantly up-regulated during the senescence process in the absence of p21. | (102) |
| miR-125b | upregulated | 20 pairs of tumor tissues and ANCTs | HEK293T, HepG2 and Sk-hep-1 cells | SIRT6 | _ | ↑↑ miR-125b: ↑ cellular senescence and apoptosis of HCC cells via targeting SIRT6 | (103) |
|  | upregulated | 125 patients with low grade glioma and glioneuronal tumors | Pediatric glioma cell lines Res186, Res259, UW479 and BT66 | _ | _ | ↑↑ miR-125b: ↑ senescence in BRAF-KIAA1549 PA cells and apoptosis in pediatric low grade glioma lines (PLGG), and ↓ growth and invasion | (104) |
| miR-126-3p | upregulated | 30 treatment-naïve Fabry patients and 60 healthy controls | HUVECs | _ | _ | ↑↑ miR-126-3p: ↑ senescence of HUVECs in Fabry disease  miR-126-3p contained in extracellular vesicles was found to be considered as a premature aging biomarker. | (105) |
| miR-126 | downregulated | _ | HUVECs | HIF-1α | _ | ∆ miR-126: ↓ HIF-1α expression protein in endothelial cells  Levels of miR-126 are decreased in senescent endothelial cells. | (106) |
|  | downregulated | _ | human glomerular mesangial cells (HGMCs) | _ | Akt-p53-p21 signaling pathways | High glucose treatment: ↑ premature senescence of HGMCs and expression of p53, p21 and Rb proteins and ↓ expression of miR-126 and p-Akt  Tea polyphenols: ↓ high glucose-induced senescence of HGMCs via miR-126 and Akt-p53-p21 signaling pathways | (107) |
|  | downregulated | _ | human glomerular mesangial cells (HGMCs) | _ | JAK/STAT signaling pathway activity | ↑↑ miR-126: ↓ senescence of HGMCs induced by high glucose, telomere-p53-p21-Rb and JAK/STAT signaling pathway activity | (108) |
|  | downregulated | _ | human glomerular mesangial cells (HGMCs) | _ | telomere-p53-p21-Rb signaling pathway | High glucose (HG) treatment: ↑ senescence of HGMCs and expression of p53, p21 and Rb proteins by activating the p53-p21-Rb signaling pathway  Tea polyphenols: ↓ high glucose-induced senescence of HGMCs via and Akt-p53-p21 signaling pathways ↓  p-STAT3, p53, P21 and Rb induced by HG, ↑ miR-126 | (109) |
| miR-126a | downregulated | male C57BL/6J  mice | HEK293A, HEK293T, BMSC, HeLa, NIH 3T3, and 3T3-L1 | VCAN | NF-κB pathway activation | ∆ miR-126a: ↑ hepatocyte senescence, inflammation, metabolism deficiency, and severity of liver defects  VCAN (a target of miR-126a-5p) promotes telomere shortening, BMSC senescence via NF-κB pathway activation. | (110) |
| miR-127-3p | upregulated | _ | mouse myofibroblasts | _ | _ | ↑↑ miR-127-3p (as an epigenetic activator): ↑ prolonged cell cycle arrest with unique molecular features of senescence, β-galactosidase, p53 and p21 levels and ↓ proliferation | (111) |
| miR-129-3p | upregulated | male C57BL/6J mice | HEK293T, human colon carcinoma cell lines HCT- 8 and HCT-116, and normal colon epithelial cell line FHC | Pirh2, p53 | _ | AVN A treatment: ↑ miR-129-3p so ↑ cellular senescence in human colon cancer cells via Pirh2/p53 pathway | (112) |
| miR-130a-3p | downregulated | male db/db mice and male db/m control mice | HK-2 human renal proximal tubular epithelial cell line | MBNL1, STAT3 | _ | Metformin via MBNL1/miR-130a-3p/STAT3 pathway decreased the senescence of renal tubular epithelial cells in diabetic nephropathy.  Metformin increases MBNL1 levels and MBNL1 prolongs the half-life of miR-130a-3p, miR-130a-3p targets STAT3, thus reduces the senescence of renal tubular epithelial cells. | (113) |
| miR-130a | downregulated | C57Bl/6 mice | Human umbilical vein endothelial cells (HUVECs), and Mouse aortic endothelial cell (MAEC) | MEOX2 and HOXA5 | _ | ∆ miR-130a: ↑ senescence and ↓ angiogenic functions  ↑↑ miR-130a: ↓ senescence and ↑ angiogenic functions in old endothelial cells and  ↑ number and improved functional activities of pro-angiogenic cells (PACs) via targeting MEOX2 and HOXA5 | (114) |
| miR-130b~301b Cluster | upregulated | 125 prostate tissue samples | LNCaP cells and DU145 cells | _ | _ | ↑↑ miR-130b~301b  Cluster: ↑ senescence-associated secretory phenotype and ↓ malignant phenotype of prostate cancer cells. | (115) |
| miR-138 | upregulated | 10 Pairs of HCC tissues (5 HCV-associated HCC patients and 5 HBV-associated HCC patients) and ANCTs | Huh7 and HepG2 cells | TERT | _ | miR-138 increases HCC cell senescence via targeting TERT, however HCV core protein suppresses miR-138, consequently decreases HCC cell replicative senescence. | (116) |
| miR-139-5p | upregulated | BALB/c mice | primary osteoblasts from BALB/c mice, Vascular endothelial cells | TBX1 | _ | ↑↑ miR-139-5p: ↑ senescence and apoptosis of vascular endothelial cells and ↓ proliferation and migration via targeting TBX1  High levels of miR-139-5p were found in senescent osteoblasts and their exosomes that regulate endothelial cell function via exosomal pathway. | (117) |
| miR-141/miR-146b-5p | upregulated | _ | HFSN1 (primary normal human skin fibroblast) | p16 ^INK4A^, AUF1 | _ | p16 ^INK4A^ reduces epithelial-to-mesenchymal transition and promotes senescence via microRNA-141/microRNA-146b-5p-dependent repression of AUF1. | (118) |
| miR-141-3p | upregulated | _ | stem cells from apical papilla (SCAPs) | YAP | _ | ↑↑ miR-141-3p: ↑ senescence of SCAPs, and ↓ proliferation of SCAPs via targeting YAP | (119) |
| miR-142 | upregulated | male C57BL/6N mice | bone marrow mesenchymal stem cells (BMMSCs) | Epas1 | _ | ↑↑ miR-142: ↑ ROS accumulation via disruption of selective autophagy for peroxisomes | (120) |
| miR-145 | upregulated | male C57BL/6J mice | human HSC line (LX‐2) and rat HSC cell line HSC‐T6 | ZEB2 | p53 pathway | ↑↑ miR-145: ↑ the number of senescence-associated β-galactosidase-positive HSCs via ZEB2/p53 pathway | (121) |
|  | upregulated | 17 T2DM patient and 19 non-diabetic patients | Smooth Muscle Cell (SMC) from non-diabetic patients (ND-SMC), or patients diagnosed with type 2 diabetes (T2DM-SMC) | _ | _ | ↑↑ miR-145: ↑ senescence, and activation of chronic stress signaling in response to DNA damage , and ↓ cell proliferation | (122) |
| miR-145a | upregulated | C57BL/6 mice | primary mouse bone marrow MSCs (mBMSCs) from femurs and tibias of C57BL/6 mice and C3H10T1/2 cells | Cbfb | P53 pathway | ↑↑ miR-145a: ↑ cellular senescence and ↓ osteogenic differentiation in MSCs via targeting Cbfb | (123) |
| miR-146a-5p | upregulated | visceral adipose tissue of df/df mice and cultured pre-adipocytes | 3T3-L1 cells and 3T3-L1 HG cells | _ | _ | ↑↑ miR-146a-5p: ↑ cellular senescence and inflammation and ↓ pro-apoptotic factors in visceral adipose tissue of df/df mice and in 3T3-L1 cells cultivated at normal but not high glucose levels  miR-146a-5p shows a different role in healthy versus stressed cells. | (124) |
| miR-146a | upregulated | ApoE-/- and wild-type (wt) C57BL/6J mice/ female C57BL/6J mice | Lineage-negative bone marrow cells (lin- BMCs) from wt or apoE–/– C57BL/6J mice | Plk2 | p16Ink4a/p19Arf and p53 pathway | ↑↑ miR-146a: ↑ senescence and apoptosis via p16Ink4a/p19Arf and p53 in young lin- BMCs and ↓ Plk2 expression and angiogenic capacity in vitro and in vivo | (125) |
|  | downregulated | 10 SLE patients and 10 healthy controls | BM-MSCs | _ | TRAF6-NF-κB Signaling | ↑↑ miR-146a: ↓ SLE MSCs senescence via targeting TRAF6 | (126) |
| miR-155-5p | upregulated | female C57/B6J mice | Human YMSCs and AMSCs and 293T cells | Cab39 | AMPK signaling pathway | ↑↑ miR-155-5p: ↑ YMSCs senescence  ∆ miR-155-5p: ↓ AMSC senescence | (127) |
| miR-155 | upregulated | _ | human umbilical vein endothelial cells (HUVECs) | TNF-alpha, SIRT1 | SIRT1/FoxO-1/p21 pathway signaling | TNF-alpha-induced miR-155 targets SIRT1, thus increases senescence and decreased proliferation in HUVECs. | (128) |
| miR-183-5p | upregulated | male C57BL6 mice | primary bone marrow stromal cells (BMSCs) from the femora of C57BL/6 mice | Hmox1 | _ | ↑↑ miR-183-5p: ↑ cell senescence and ↓ proliferation and osteogenic differentiation | (129) |
| miR-185 | upregulated | _ | HTC75, HEK293T and MRC5 cell lines | POT1 | POT1-ATR signaling pathway | miR-185 induces cell senescence by targeting POT1. | (130) |
| miR-188 | upregulated | _ | Lineage-negative bone marrow cells (lin-BMCs) | MAP3K3 | _ | ↑↑ miR-188: ↑ cell senescence, and ↓ cell proliferation, migration, and tube formation via targeting MAP3K3 | (131) |
| miR-194 | upregulated | _ | primary mouse embryonic fibroblasts (MEFs) from C57BL/6 mouse and an immortalized mouse embryonic fibroblasts (NIH/3T3), and a SV40-transformed human embryonic kidney cell line (HK293T)/  human umbilical vein epithelial cells (HUVECs) | DNMT3A | _ | ↑↑ miR-194: ↑ the replicative senescence of MEFs and H2 O2 -induced senescence of NIH/3T3 cells via targeting DNMT3A so increases development of cellular senescence in mouse embryonic fibroblasts | (132) |
|  | upregulated | male C57BL/6 mice | ethanol-induced LO2 cells | FXR | _ | Yan decreased chronic ethanol-induced liver injury by inhibition of cellular senescence via suppressing miR-194 and increasing levels of FXR. | (133) |
| miR-197-5p | upregulated | _ | HT1080 fibrosarcoma cells and MR90-tert fibroblast cells | KIAA0101 | _ | ↑↑ miR-197-5p: ↑ cellular senescence and ↓ viability and proliferation of fibrosarcoma cells via targeting KIAA0101 | (134) |
| miR-199a-5p | upregulated | Abdominal aortic tissue from AAA patients and healthy aortic tissue from controls | Vascular smooth muscle cells (VSMCs) from AAA patients | SIRT1 | _ | ↑↑ miR-199a-5p: ↑ Angiotensin II-induced VSMC senescence by targeting SIRT1 | (135) |
|  | upregulated | C57BL/6J male mice | Adipose-derived MSCs from 7 healthy controls and 6 IPF patients (control-MSCs and IPF-MSCs) | SIRT1 | AMPK signaling pathway | ↑↑ miR-199a-5p: ↑ senescence of control-MSCs, and ↓ autophagy of MSCs via the AMPK signaling pathway via targeting SIRT1  ∆ miR-199a-5p: ↓ IPF-MSC senescence | (136) |
| miR-199a-5p | upregulated | female MRL/lpr and ICR mice | human umbilical cord-derived mesenchymal stem cells (hUC-MSCs) | _ | Sirt1/p53 pathway | ↑↑ miR-199a-5p: ↑ splenic CD4+ T-cell senescence and the therapeutic effects of transplanted hUC-MSCs in lupus | (137) |
| miR-200 | downregulated | TCGA database and GEO database: (GSE62254)/  female NSG mice | human GC cell line AGS | _ | _ | ∆ miR-200: ↑ EMT associated cellular senescence in gastric cancer (GC) | (136) |
| miR-200a | upregulated | _ | primary keratinocyte from biopsies of the skin from 20 healthy donors and human embryonic kidney (HEK) 293 cells | OGG1-2a,  Bmi-1 | _ | ↑↑ miR-200a: ↑ senescence in aged keratinocytes via targeting OGG1-2a | (138) |
| miR-200b | downregulated | COPD model mice | mouse lung epithelial cell line MLE-12 | ZEB2 | _ | miR-200b could inhibit inflammatory responses and cellular senescence by targeting ZEB2 in pulmonary emphysema. | (139) |
| miR-200c | upregulated | C57/BL6 mice | normal human keratinocytes (NHKs) | _ | _ | ∆ miR-200c: ↓ cell senescence, inflammation in NHKs exposed to IR and ↑ cell proliferation in NHKs after irradiation, migration and EMT process in RIOM | (140) |
| miR-203 | downregulated | 25 pairs of ovarian cancer tissues and ANCTs/ male nude mice | ovarian cancer cell lines OVCAR3, OVCA429 and HEK293T | ITPKA | _ | ↑↑ ITPKA (a target of miR-203): ↑ cell senescence, ↓ ovarian cancer tumorigenesis | (141) |
| miR-204 | upregulated | knee joint cartilages from OA patients with and aging-associated and posttraumatic OA mice/ OA mouse models | primary cultured human and mouse chondrocytes | _ | _ | ∆ miR-204: ↓ osteoarthritis with concomitant recovery of PG synthesis and suppression of inflammatory senescence-associated secretory phenotype (SASP) factors in cartilage | (142) |
| miR-206 | upregulated | female SD rats | Bone marrow-derived mesenchymal stem cells (MSCs) from SD rat | _ | _ | ∆ miR-206: ↓ H2O2-induced senescence and dysfunction in MSCs | (143) |
| miR-214 | upregulated | Venous blood samples were collected from 16 CAS patients | Rat aortic vascular smooth muscle cell (A7r5) | _ | _ | CoCl2 treatment: ↑ miR-214 level: ↑ cell senescence in VSMCs and ↓ cell proliferation and angiogenesis | (144) |
| miR-216a | upregulated | 176 CAD patients and 342 healthy controls | primary  human umbilical vein endothelial cells (HUVECs) | _ | Smad3/IκBα pathway | miR-216a induces endothelial senescence and inflammation via targeting of Smad3/IκBα pathway.  Levels of miR-216a were higher in old CAD patients compared with the healthy controls. | (145) |
|  | upregulated | _ | human umbilical vein endothelial cells (HUVECs) and human aortic endothelial cells | VCAM1 | Smad3/NF‑κB signaling pathway | ↑↑ miR‑216a: ↑ premature senescent‑like phenotype  Ginsenoside Rb2 treatment: ↓ miR‑216a levels, senescence, and inflammation | (146) |
| miR-217 | upregulated | Endothelial-specific inducible miR-217 C57BL/6J knock-in mice | Lung endothelial cells from wild-type C57BL/6J mice, primary human vein endothelial cells | _ | _ | miR-217 promotes atherosclerosis and aggravates cardiovascular dysfunction by increasing aging and cellular senescence. | (147) |
|  | upregulated | _ | HEK 293T cells | DNMT1 | _ | ↑↑ miR-217: ↑ senescence phenotype in young fibroblasts via targeting DNMT1 | (148) |
| miR-221 | downregulated | _ | Human lung cancer cell lines H1299, H226, and A549 | _ | PTEN/Akt pathway | ∆ miR-221: ↑ senescence and ↓ proliferation in lung cancer cells  MiR-221, MDR1 and ABCG2 were upregulated in Cisplatin-resistant A549 lung cancer cells. | (149) |
| miR-222 | upregulated | _ | breast cancer cell lines MDA-MB-231 and MDA-MB-157 and NF and CAF fibroblast lines from breast cancer samples | LBR | _ | ↑↑ miR-222: ↑ NFs to show the CAF characteristics of enhanced migration, invasion and senescence | (150) |
| mmu-miR-291a-3p | downregulated | _ | mouse embryonic stem cells (ESCs) | _ | TGF-β Receptor 2 Pathway | ↑↑ mmu-miR-291a-3p: ↓ senescence-associated β-galactosidase activity, mRNA and protein expression of TGF-β receptor 2, p53, and p21 and ↑ proliferation | (151) |
| miR-300 | downregulated | _ | Human non-small cell lung cancer cell line A549, kidney cancer cell line 786-O, breast cancer cell line MCF7 and melanoma cell line A375, Human non-small cell lung cancer cell line GLC82 and small cell lung cancer cell line NCI-H446 (H446), NCI-H1299 (H1299), human melanoma cell lines 92-1 and OCM-1, H1299 | p53, apaf1 | _ | ↑↑ miR-300: ↓ sensitivity lung cancer cells to IR via inhibiting p53-dependent G2 cell cycle arrest, apoptosis and senescence | (152) |
| miR-302b-3p | upregulated | aged skin tissues and young control tissues/  BALB/c female mice | _ | SIRT1, JNK2 | _ | ↑↑ miR-302b-3p: ↑ senescence fibroblasts via directly targeting JNK2  ∆ miR-302b-3p: ↓ skin fibroblast senescence | (153) |
| miR-326 | downregulated | olfactory mucosal tissue /  male C57BL/6 mice | Human OM-MSCs | PTBP1 | _ | ↑↑ miR-326: ↓ normoxia OM-MSC senescence  ∆ miR-326: ↑ hypoxia OM-MSC senescence by upregulating autophagy via targeting PTBP1 | (154) |
| miR-340-5p | downregulated | AD mouse model | HT22 cells | POT1 | _ | ↑↑ miR-340-5p: ↓ cellular senescence and AD symptoms and ↑ cellular telomere length via targeting POT1 | (155) |
|  | upregulated | _ | WI-38 human diploid fibroblasts, primary human coronary artery vascular smooth muscle cells (VSMCs) and H9C2 cells | LBR | _ | ↑↑ miR-340-5p: ↑ cell senescence, senolysis, and disruption of chromatin by targeting LBR | (156) |
| miR‑342‑5p | downregulated | Zmpste24−/− and wild-type (WT) mice | primary MEFs from embryonic day (E) 13.5 embryos of *Zmpste24*−/− and wild-type (WT) mice | GAS2 | _ | ↑↑ miR‑342‑5p: ↓ cellular senescence phenotype, ↑ cell proliferation and ↑ the G2+M cell cycle phase in Zmpste24‑/‑ MEFs via targeting GAS2 | (157) |
| miR-363-3p | upregulated | _ | Bone marrow-derived mesenchymal stem cells (BMSCs) from rats | TRAF3 | _ | ↑↑ TRAF3 (a target of miR-363-3p): ↑ BMSC osteogenic differentiation and ↓ adipogenic differentiation and senescence | (158) |
| miR-384-5p | upregulated | Sprague‐Dawley rats | BMSCs from young and aged rats | Gli2 | _ | ↑↑ miR-384-5p: ↑ senescence and ↓ osteogenic differentiation in young BMSCs via targeting Gli2  ∆ miR-384-5p: ↓ senescence and ↑ osteogenic differentiation in aged BMSCs | (159) |
| miR-483-3p | upregulated | _ | Human adipose-derived mesenchymal stem cells (hADSCs) | IGF1 | _ | ∆ miR-483-3p: ↓ cellular senescence and adipogenic differentiation potential of hADSCs | (160) |
| miR-494-3p | upregulated | Lung tissues from 8 nonsmoker and 8 non- COPD smokers, 16 moderate/mild COPD patients, and 12 severe COPD patients | small airway epithelial cells (SAECs) and BEAS-2B cells | SIRT3 | p38MAPK-c-myc signaling pathway | ↑↑ miR-494-3p: ↑ cellular senescence and senescence-associated secretory phenotype via targeting SIRT3 in COPD  MiR-494-3p expression is induced by oxidative stress through the p38MAPK-c-myc signaling pathway. | (161) |
| miR-503 | downregulated | _ | human pancreatic epithelial cell lines HPNE, BxPC-3 | ARID1A and CDKN2A | _ | ∆ ARID1A (which represses miR-503): ↓ KRAS^G12D^-induced cell senescence, ↑ cell proliferation and colony formation in cooperation with active mutant KRAS^G12D^ through miR-503/CDKN2A axis | (162) |
| miR-570-3p | upregulated | Lung tissues from COPD patients and matched controls | BEAS-2B cells | sirtuin-1 | _ | ∆ miR-570-3p: ↓ cellular senescence by restoring the antiaging molecule sirtuin-1 in COPD | (163) |
| miR-623 | downregulated | intervertebral disc degeneration tissues | Nucleus pulposus cell (LPS-injured NPCs) | CXCL12 | _ | ↑↑ miR-623: ↓ cell apoptosis, senescence, inflammation, and ↑ ECM secretion via targeting CXCL12 | (164) |
| miR-665 | upregulated | _ | Human VMSCs | SDC1 | _ | Levels of miR-665 are upregulated in response to BLM-induced VSMC senescence because of aberrant DNA methylation.  ↑↑ miR-665: ↓ effects of OMST on BLM-induced VSMC senescence via targeting SDC1 | (165) |
| miR-675 | downregulated | C57BL6 mice | H9C2 cells | TGF-β1 | _ | miR-675 which is delivered by stem cell-derived exosomes encapsulated in silk fibroin hydrogel reduces aging-induced vascular dysfunction in mouse hindlimb via targeting TGF-β1. | (166) |
| miR-1202 | _ | Gene expression profile of GSE105027, (12 osteoarthritis  (OA) patients and 12 healthy controls | _ | KCNQ1OT1, ETS1 | _ | miR-1202 regulates histone acetyltransferase pathway binding function and cellular senescence pathway, thus regulate OA development via KCNQ1OT1-has-miR-1202-ETS1 axis. | (167) |
| miR-1299 | downregulated | 6 pairs of melisma tissues and ANCTs | Keratinocytes and Melanocytes | ARG2 | _ | ↑↑ ARG2 (a target of miR-1299): ↓ autophagy in keratinocytes by inducing cellular senescence | (168) |
| miR-1468-3p | upregulated | Samples of sudden cardiac death (SCD) victims with primary myocardial fibrosis (PMF) and healthy myocardial  controls | hCFs | DUSP1 | TGF-β1-p38 signaling | ↑↑ miR-1468-3p: ↑ cellular senescence with high senescence-associated β-galactosidase activity and high expression of p53 and p16 | (169) |
